# Identifying Policy Best-Practices to Support the Contribution of Aquatic Foods to Food and Nutrition Security

**DOI:** 10.3390/foods10071589

**Published:** 2021-07-08

**Authors:** Anna K. Farmery, Amy White, Edward H. Allison

**Affiliations:** 1 Australian National Centre for Ocean Resources and Security, University of Wollongong, Wollongong 2522, Australia; 2 Impacto, Melbourne 3000, Australia; amy.white@impact-o.com.au; 3 WorldFish, Penang 11960, Malaysia; E.Allison@cgiar.org

**Keywords:** aquatic foods, fisheries, aquaculture, health, governance, food systems

## Abstract

The relationship between aquatic foods and food nutrition and security is increasingly recognised in policy and practice, yet many governance instruments do not acknowledge or support this important connection. The most effective policy approaches to support the link between these sectors, or ‘best practices’ are currently unknown. We reviewed relevant governance instruments from multiple countries to identify how these instruments linked fisheries, aquaculture and food security and nutrition, including the policy framing and evidence of political commitment. Of the documents connecting the sectors (65%), the majority did so in the context of developing the fisheries/aquaculture sector to increase aquatic food availability and/or access (51%), followed by developing the fisheries/aquaculture sector as a livelihoods approach to indirectly improve food security (33%), for example, through income generation. Sectoral links established in the context of nutrition-sensitive approaches to fisheries and aquaculture were less common (5%). Almost one third (29%) of instruments supported the connection between aquatic foods and food security and nutrition across three or more different contexts relevant to food security or food systems, while 12% indicated a very high level of commitment. We recommend some key attributes for future policy development to help build coherence between sectors and to help frame coherent food system-based policies.

## 1. Introduction

A growing body of research highlights the important contribution of aquatic foods to food and nutrition security (FNS) and to sustaining healthy diets [1,2,3,4,5,6]. However, the contribution of wild-capture fisheries and aquaculture remains underrepresented in major FNS policy initiatives [3,7] and sustainable diet research [8]. Aquatic foods—plants and animals produced through aquaculture and wild-capture fisheries—help to overcome hunger and malnutrition by providing bioavailable micronutrients and protein, as well as income for fishers, processors, traders and sellers to purchase food [9]. The production of food, including aquatic foods from fishing and aquaculture, can result in environmental impacts, such as ecosystem degradation [10] and greenhouse gas emissions [11,12]. However, aquatic foods have substantial potential to improve global diets, as a highly nutritious source of food that can have a lower environmental footprint than other animal-sourced proteins [13,14]. Aquatic foods contribute variously to the four pillars of food security—availability, access, utilisation and stability—in which nutrition is integral [15] and ongoing support is required to ensure their contribution in order to meet a range of Sustainable Development Goals (SDGs) [2].

The FAO has developed a policy guidance note to assist countries in strengthening fisheries and aquaculture sector policies for better FNS results [16] and many countries have identified the contribution of aquatic food to FNS in their national governance instruments. Despite these efforts, policy recognition and support for the role of aquatic foods in human diets remains relatively low [17]. Where recognition does exist, the extent to which it will translate into effective action is not yet known. Monitoring and measuring the impact of these polices is limited, in part, by the lack of information on FNS issues in the fisheries and aquaculture sectors [16] as well as by the deficiency of reliable FNS indicators across all pillars [18,19], in particular those suitable for aquatic foods.

Greater political commitment is needed to link fisheries and aquaculture with food security and public health [16,20,21]. In addition, there is a need to reframe policies to establish fish as food (in addition to a trade commodity, or an environmental governance challenge) [2,4] and to support the role of aquatic foods within a ‘food system’ to address concerns around health, sustainability and equity in food access, affordability and consumption [20,21,22,23]. Consideration of these broader food system elements is gaining traction in agricultural research and policy, although the role and contribution of aquatic foods in food systems has largely been ignored [24,25].

As more countries articulate links between aquatic foods and FNS within their national policy frameworks, identifying and sharing current ‘best practices’ can be a useful approach to assist policy development. There is no single definition of policy ‘best practice’, but the term is generally used to identify policies that are considered to be successful and useful for assisting other entities grappling with similar challenges [26]. Best practices, once identified, have traditionally been used as blueprints to replicate good policies and propel effective policy-making and change [27]. However, caution should be exercised when attempting to replicate polices, as what is considered good in one context is not necessarily so in another [28]. The best practices concept can instead be used to enable or provoke stakeholders and politicians to improve policies, and to frame what is doable or desirable in order to assist decision-making [29]. In this context, the concept of best practice is examined here with the intention of identifying potential tools to learn from, frame debates and identify possibilities and not to replicate a blueprint [26].

Framing is an important aspect of the policy development process [30,31] as the framing of issues determines policy actors’ priorities and course of action [32]. Reframing policies to support aquatic foods as part of food systems will also require a degree of political commitment to be effective. Food and nutrition historically have been given low political priority and suffered from lack of commitment from national governments. Building political commitment is, therefore, necessary to progress food and nutrition security on government agendas [33]. This review of existing policies aims to identify elements of best practices that will help reframe policy-making toward a more coherent food systems-based approach that is inclusive of aquatic foods, as well as demonstrative of political commitment. To achieve this aim, we examine public governance instruments (frameworks, laws, policies, plans, programmes and strategies) related to fisheries, aquaculture and FNS, to understand current approaches relating to the support of the connection between aquatic foods and FNS, and to develop recommendations for future policy development. We determine, firstly, the existence of linkages between aquatic foods and FNS within the instruments; secondly, the policy framing based on the contexts of the linkages within an instrument; and thirdly, the inferred level of political commitment to achieving policy goals. The results provide insights into the approaches taken by different countries to link aquatic foods with FNS and examples of current policy framing that is supportive of a food systems approach. The dynamic character of research on aquatic foods and food systems means that what is considered best practice will likely change quickly. The findings and recommendations presented in this paper, therefore, represent what could be considered the best practices in 2021.

## 2. Materials and Methods

To address our research question—what are the policy best practices for supporting the contribution of aquatic foods to FNS?—we examined fishery, aquaculture, and FNS-related governance instruments from a selection of countries. The stages of research, detailed below, include (a) the selection of case study countries, (b) identification of governance instruments relevant to fisheries, aquaculture and/or FNS, (c) document key word search, and (d) analysis of current best practice. This last stage involved characterising the ‘linkages’ between fisheries/aquaculture and FNS into contexts that reflect different policy framings, and assessing how these support a broader food system framing, as well as examining the inferred level of political commitment, which is critical for moving policy in the desired direction.

### 2.1. Selection of Case Study Countries

To identify countries for inclusion in the analysis, a list was compiled of countries which potentially offered examples of best practices, based on national aquatic food production and the importance of aquatic foods for national FNS. This approach was not intended to provide statistical representativeness, rather, the countries were selected based on the assumption that they were more likely to have governance instruments linking aquatic foods with food and nutrition and it was these instruments that we wanted to identify to undertake the analysis. The initial list of 44 countries included the world’s 30 largest aquatic food-producing countries (based on FAO production data 2009–2018), and fourteen countries identified by the research team where the population is reliant on aquatic foods for a significant portion of their intake of animal protein or other key nutrients [34,35].

The second step to select the countries was undertaken using the FNS and fisheries policy database developed by Koehn et al. [17], which scored countries based on the degree of linkages between national policies related to fisheries and FNS. Koehn’s policy linkage analysis was a two-way process, i.e., it examined and scored the extent to which FNS was considered in national fisheries and aquaculture policies, and the extent to which aquatic foods were represented in food security and nutrition-related national plans and policies. We used the policy-linkage score from Koehn to identify countries (from the original list of 44) that scored three or above (with four being a high score showing strong linkage) for fisheries and/or nutrition (Table 1).

A total of 14 countries were identified. From this list, the United States of America was excluded and replaced with Senegal, based on the greater importance of aquatic food for FNS in this major West African fishing nation. Indonesia was also added to the final list. This country had a low score at the national level in the Koehn database, probably because the government has recently shifted responsibility for fisheries to the provincial level in response to the problems associated with the complex mix of law, regulations and decrees at the national level, and Koehn’s analysis was confined to national policies. Indonesia was added to this study to provide a case study of a provincial-based approach to fisheries policy (Table 1).

### 2.2. Identification of Relevant Governance Instruments and Key Word Search

The FAOLEX database (FAO, 2020) was used as the primary search engine to find relevant national governance instruments for the selected countries. A list of key search terms was developed based on key issues identified in the literature on aquatic foods and food and nutrition security. The term ‘aquatic foods’ is relatively new and has been adopted by organisations including WorldFish to ensure that the fisheries and aquaculture sectors are treated as one and are inclusive of foods such as seaweeds and marine mammals, for example, and not only fish and shellfish [36]. It was not expected that this term would be found in the documents examined. Instead, key words were identified that related to the wild capture and aquaculture systems that produce aquatic foods. Search terms were translated to Indonesian, Spanish and French by bilingual members of the research team (Tables S1 and S2). These terms were used to identify the links between fisheries/aquaculture and FNS in the governance instruments. Searches were conducted using a filter for polices and laws relating to food and nutrition as well as fisheries and aquaculture. In some cases, FAOLEX identified the key search terms in governance instruments from additional sectors, such as coastal development, climate change or sustainability, or within multi-sectoral instruments such as strategic development plans. A separate search was conducted using Google search engines to locate laws that related to fisheries/aquaculture and FNS. Documents that were not in English were translated using a combination of Google Translate and bilingual members of the research team.

For Indonesia, relevant instruments were sourced from the provincial government websites of Central Java and West Java. These provinces were selected for inclusion based on several considerations. First, they had comprehensive strategic planning documents, in comparison to other provinces, which suggested increased potential for connections between food security and aquatic food objectives. Second, these provinces are coastal, and aquatic food consumption is high and expected to increase, in part as a result of campaigns to increase fish consumption. For example, the “gemar makan ikan” (enjoy eating fish) movement is a program from the Ministry of Marine Affairs and Fisheries. Third, a high percentage of the population in these provinces depend on fishery activities for their livelihoods.

We note that our results do not capture all policies from all countries that will be relevant to aquatic foods and FNS. Our approach was to try and capture as many relevant policies as possible from countries that are known to have strong aquatic food sectors or where aquatic foods are important for FNS, with the assumption that these countries may be more likely to have established policies linking the sectors, and, therefore, be informative in examining and identifying best practices. What is also of note is that our approach does not accommodate the hierarchy of domestic legislation and policy. Instead, national policy is assessed against state or provincial law, policy or actions plans. This type of analysis is common but makes the assumption that the documents are equal. The presence of a law linking fisheries/aquaculture and food and nutrition was not assumed to indicate a strengthened commitment to policy implementation.

### 2.3. Criteria for Identifying Best Practices

As outlined above, the concept of best practice is used here in order to identify potential tools to learn from, frame debates, and identify possibilities, and not to replicate a blueprint [26]. Identifying policy best practices can help frame what futures are desirable [27] and our examination focusses on current policy framing of aquatic foods and the best practices available for developing coherent food system-based policies that link aquatic foods with healthy, fair and sustainable outcomes. Identifying best practices in any situation inevitably involves judgement. To identify best practices for linking aquatic foods and FNS, within a food system policy frame, we established key criteria against which the policies were assessed. In this research, where we aimed to identify what policy-framing best-practices currently look like, we propose that policy documents should at least:

- Establish a link between aquatic food and FNS (described above in Section 2.2);
- Establish links between aquatic food and FNS within multiple contexts that address a range of food security pillars or food system elements;
- Demonstrate a high to very high level of political commitment in terms of institutional or budgetary support;

### 2.4. Context of Link between Aquatic Food and Food Security and Nutrition

For each instrument in which a link between aquatic foods and FNS was identified, a more detailed examination was undertaken to understand the policy framing or context of the link. A range of different contexts were identified in the governance documents. These were grouped into key or dominant themes based on common themes identified in the governance instruments that reflected themes in the literature on aquatic food, FNS, and food systems (Table 2). Those instruments that made linkages across multiple contexts were assumed to be more comprehensive and more closely reflected a food system framing than those that linked the sectors within a single context. The rationale for this assumption is based on (a) the multi-dimensional nature of both food security and food systems; (b) ongoing criticism of the narrow research and policy focus on single aspects of food security, such as increasing production, without consideration of how the food will be distributed and accessed [4,23,37]; and (c) the need for a nutrition-sensitive approach across aquatic food production, distribution and consumption [5,38].

The range of contexts within which the linkages between fisheries/aquaculture and FNS were made in the governance instruments was one of two criteria used to identify best practices.

### 2.5. Level of Political Commitment

The second criteria was the level of commitment demonstrated by national governments to link aquatic foods and FNS, including declarations of support within policies, any organisational infrastructure identified, or earmarked allocations of resources relative to a particular benchmark [33]. Since it was not in the scope of this research to ground truth policy commitment or effectiveness for such a large number of instruments, a proxy was used to assess the level of commitment based on the classifications shown in Table 3. It is important to note that this assessment was not undertaken for each individual reference to FNS, fisheries or aquaculture found in the instruments (using the classifications in Table 2), but rather, it was used to assess the highest level of commitment made for at least one of the references in each instrument.

## 3. Results

A total of 110 documents were reviewed for this research, from a range of sectors. Fisheries was the most commonly represented sector (29%), followed by nutrition (11%), aquaculture (11%) and agriculture (11%). Several countries had both overarching fisheries instruments as well as separate sub-sector instruments (e.g., for specific species or artisanal fisheries), which increased the total number of fishery documents. A summary of the sectors for all documents can be found in Table 4; however, it is important to note that many of the documents were multi-sectoral and/or multi-dimensional in nature and these classifications cover only the primary focus.

### 3.1. Linkages between Aquatic Foods and Food and Nutrition Security

The links established between aquatic food and FNS were identified predominantly in national policies, strategies and plans, rather than in legislation. Only 20% of the 21 laws reviewed established a link, all of which were stated in the general aims of the document. This contrasts with the 89 policy and strategy documents of which 76% articulated a link, with varying degrees of commitment (Table S3). This finding may be due, in part, to the contemporary nature of the policy and strategy documents in relation to the laws. When looking specifically at the fisheries, aquaculture and FNS sector instruments, as opposed to other sectoral instruments, the linkage between aquatic foods and FNS was more common in the fisheries or aquaculture instruments, with 80% of the instruments reviewed from this sector (excluding laws) linking aquatic food and FNS, compared to 67% of the FNS instruments. Over one third (35%) of the documents examined made no link between aquatic foods and FNS (Table 5).

### 3.2. Context of Linkage

Of the documents that established a link between aquatic foods and FNS, 65% identified a connection between sectors within a single context, or frame, and almost one third (29%) made the connection across three or more contexts relevant to food security or food systems (Table 5). The most common context in which aquatic food and FNS were connected was developing the fisheries/aquaculture sector to increase aquatic food availability and/or access (51%, *n* = 56). Food availability and access were grouped for this analysis, although many of the objectives in this context focussed on improving availability through increasing production rather than on improving access. Indicators used to measure the success of objectives to improve food security focussed predominantly on production or economic based metrics. For example, the Vanuatu National Fisheries Sector Policy 2016–2031 set a clear objective to improve food security through investment in fisheries and economic growth and included actions such as the development of new infrastructure (e.g., wharf, processing facilities), the provision of fishing gears and training for fishers and reducing the barriers to investment. The metrics used to assess progress included the completion of infrastructure projects, the number of licences issued and the export of frozen and fresh fish from the new onshore facilities. The Indian National Policy on Marine Fisheries, 2017 also mentioned the importance of reducing post-harvest losses as a way to increase availability of fish for human consumption. Several policies included objectives to improve access to aquatic food through, for example, establishing local co-ops and investing in cold chain infrastructure, although few instruments included activities that were directly related to affordability of aquatic foods.

The second most common context was developing the fisheries/aquaculture sector as a livelihood approach to improve food security (33%, *n* = 36), for example through income generation. In many cases, objectives relating to increasing productivity were also linked to improving incomes, for example Key outcome 2 of The Strategy for the Development of Samoa 2016–2020 was to increase agricultural and fisheries productivity to increase food, nutrition and income security. The context with the least links (5%, *n* = 6) was support for a nutrition-sensitive approach to fisheries/aquaculture. The nutrition-sensitive approach to production was identified in policies in Bangladesh (National Nutrition Policy 2015, Perspective Plan of Bangladesh 2010–2021, Second Country Investment Plan (2016–2020)), Ghana (National nutrition Policy 2013–2017, Medium term Agricultural Sector Investment Plan II 2014–2017) and to a lesser extent Tanzania (National Multisectoral Nutrition Action Plan 2016/17–2020/21).

Connecting fisheries/aquaculture and FNS in the context of resilience to environmental threats and resource sustainability was identified in almost a third of documents reviewed, in particular with reference to climate change and sustainable stock management. However, the connection between aquatic foods and resilience was not limited to the fisheries/aquaculture or environment sector instruments and was identified across a range of sectoral instruments including agriculture, nutrition, economic development and sustainable development (Table S3). The context of equitable and fair allocation of resources and distribution of benefits to improve food security and/or improve livelihoods was identified in 12% of documents. Whilst some policies mentioned equitable access in their general fisheries instruments, this framing often occurred within sub-sector instruments which enabled distinction between links relating to aquatic food destined for local consumption and those for export. For example, Samoa had a tuna-specific instrument which focussed on exports and the equitable allocation of resources. Both South Africa and Peru had separate instruments that focussed on small-scale/artisanal fisheries (Policy for the Small-Scale Fisheries Sector in South Africa 2012, National Plan for Development of Artisanal Fishing 2004) which had a strong focus on direct food security and equitable allocation of resources.

Objectives to increase consumption of aquatic food was articulated in 14% of documents. For example, the Tanzanian National Multisectoral Nutrition Action Plan 2016/17–2020/21 identified the need for the Ministry for Agriculture, Livestock and Fisheries as well as the private sector to invest in the marketing of high-value nutritious and healthy products (including aquatic food) as a means to improve the nutritional status of the Tanzanian population. It also encouraged a multi-sectoral approach to nutrition education starting from childhood to address nutrition-related issues, which included input from the agriculture/fisheries sector as well as others including climate change/environment and education. Peru’s National Plan for Artisanal Fisheries 2004 included an initiative requiring the state purchase programs (e.g., procurement departments from major ministries) to support the consumption of fish.

For the context of supporting the contribution of aquatic food to diets and/or livelihoods of vulnerable groups, the FNS sector documents identified women and children as vulnerable groups with a focus on the role of aquatic food in improving their nutritional status (e.g., Tanzania Agriculture and Food Security Investment Plan 2011–12–2020–21). In contrast, instruments from the food production sectors (fisheries, aquaculture, agriculture) tended to focus more on the direct and indirect role of fisheries/aquaculture in improving the livelihoods of rural (often poor) households and fisherfolk (e.g., Tanzanian Agricultural Sector Development Programme Phase Two 2016), with some (e.g., National Agricultural Investment Program for Food Security and Nutrition in Senegal 2018–2022) specifically including women and youth employed in fisheries and aquaculture. Support for women’s fish-based livelihoods was recognised in several recent policies examined here (for example the Government of Samoa Agriculture Sector Plan 2016–2020 and the National Aquaculture Development Strategy and Action Plan of Bangladesh 2013–2020); however, where a link between women or gender and livelihoods was identified in policies without a specific link to food or nutrition, it was not included.

Several countries supported the connection between aquatic foods and FNS through educational initiatives around healthy eating and the benefits of aquatic foods, although different policy approaches were taken by developed countries in comparison with developing countries. For example, Japan and Norway highlighted the role of school-based education, whilst in Peru the focus was on educating the general population (e.g., Peru National Plan for the Development of Artisanal Fishing 2004) and in Tanzania the focus was more specifically on education for women and children (e.g., Tanzanian National Plan for the Development of Artisanal Fishing 2004). Japan demonstrated a unique approach to education in their Basic Act on Dietary Education (Shokuiku) 2005 by encouraging collaboration between educators and farmers/fishers to provide educational opportunities for the general population to better understand the importance of human activities in food production and distribution.

### 3.3. Level of Commitment to Achieving Goals

The level of political commitment for implementing or strengthening the linkage between aquatic food and FNS was measured by the extent to which a document declared support within policies, any organisational infrastructure identified or allocations of resources relative to a particular benchmark, including the presence of objectives or targets set to measure progress. While the majority of instruments examined in the review linked aquatic food and food and nutrition security (72 out of 110), 25% demonstrated a low to very low level of commitment by declaring the link only within the general discussion (*n* = 7) or in the general aims of the document (*n* = 21). Only 12% of documents showed a very high level commitment by articulating specific objectives and defining targets, indicators or budget allocation, with a further 15% outlining objectives without targets, indicators or budget (Table 6).

### 3.4. Policy Best Practices

A range of different approaches were identified between the 15 countries included in this study regarding the policy framing that connected aquatic foods with FNS, and the political commitment to support the connection. Indonesia, Tanzania, Vanuatu, Senegal, Bangladesh and Samoa articulated links between aquatic foods and FNS in three quarters or more of their national instruments identified as relevant to the study (Table 7). In contrast, India, Norway, Chile and Japan, which are all large consumers and producers of aquatic foods, linked the sectors in 40% or less of relevant instruments. Of those countries that articulated connections between sectors across different instruments, there was variation in policy framing and political commitment. Indonesia, for example, linked aquatic food with FNS across 90% of instruments examined, although in most instruments (70%) this link was limited to two different contexts, predominantly developing the fisheries/aquaculture sector to increase aquatic food availability and/or access, and increasing aquatic food consumption. Despite the limited contexts covered within individual instruments, the majority of contexts (identified in Table 5) were addressed across all the Indonesian instruments. In Samoa, 5 out of 8 documents identified the link between sectors across multiple contexts and almost 40% had high to very high levels of commitment (Table 7). In addition, these links were made in documents from different sectors including fisheries, aquaculture, agriculture and development showing potential for increased coherence across these sectors. The National Food and Nutrition Policy 2013 was an outlier and did not link aquatic foods with FNS.

Vanuatu also demonstrated a high number of instruments linking aquatic food and FNS, with 43% linking the sectors within multiple contexts and 57% having high to very high commitment. Coherence across sectors was also positive, with the link between aquatic food and FNS established across all sectoral instruments (including fisheries, FNS, agriculture and development) except the fisheries law (Fisheries Act No 10 of 2014).

The links between aquatic foods and FNS were predominantly identified at the level of national policies, strategies and plans, rather than in legislation. With the exception of Peru and the Philippines, fisheries laws did not link fisheries management with FNS. However, establishing the link within a law did not translate into a high level of commitment to implement actions across instruments in Peru or the Philippines. The extent to which enshrining the link between aquatic food and FNS in law facilitates improved health outcomes is an area requiring further research.

## 4. Discussion

The connection between aquatic foods and food security and nutrition (FNS) is being supported through national governance instruments in a range of novel and country-specific ways. However, there are many gaps and weak points that can be strengthened, including in countries where stronger support was expected given the importance of aquatic foods for livelihoods or nutrition. Where the link between sectors was established, it was generally considered within a narrow policy frame that did not consider broader elements relating to food security or food systems. In addition, many policies lacked political commitment in the form of clear targets, actions or budgetary support. The policies that demonstrated potential for a food system framing, through connecting aquatic foods and FNS across a range of different contexts, and a high level of political commitment, can provide insights into policy best practice in this field.

### 4.1. Policy Approaches to Linking Aquatic Foods and FNS

Understanding the policy frames in which aquatic foods are being linked with FNS is an important step in assessing the potential impact of polices, as well as in identifying competing agendas across policy communities [32]. Developing the fisheries/aquaculture sector to increase aquatic food availability and/or access was the most common policy framing in which aquatic food and FNS were connected, with many objectives focusing on improving availability through increasing production, rather than on improving access. Increasing the production and global availability of food has historically been, and continues to be, the dominant focus of food security approaches [37,39]. However, this approach has been widely criticised given that over 26% of the world population remains food insecure and hunger is rising [40] despite ongoing growth in agricultural production and a global food surplus [41]. Increased production does not automatically lead to food security outcomes, as local consumers can miss out on the benefits of production where producers sell to the highest bidders, commonly in international markets [21]. Accessibility generally relates to affordability and physical access, as well sociocultural access and preferences [42]. Policies linking aquatic foods and FNS need to ensure the actions and indicators for increasing production are more clearly aligned with outcomes across the pillars of food security, including access and also consider broader food system issues [20,43].

The second most common policy framing identified was developing the fisheries/aquaculture sector as a livelihoods approach to improve food security. Malnutrition is largely seen as a reflection of poverty [44]; however, higher income is not always an appropriate proxy for food security, as income level does not always reflect the economic conditions of the household assets held, or the levels of access to other supports that prevent food insecurity [45]. In addition, the impact of increased income on nutrition outcomes is dependent on how that income is spent. Increased incomes may be spent on food that is not the most nutritious [46], or on other items than food, including detrimental items such as tobacco and alcohol.

It is now broadly accepted that income generation, on its own, is not sufficient to bring about improvements in nutrition [47]. Further research and policy development are required to better articulate the link between employment opportunities, livelihood improvement and FNS outcomes, and to set appropriate targets to measure performance, in particular to ensure that benefits reach vulnerable groups [48,49]. Developing the fisheries/aquaculture sector as a livelihood approach within a food system policy framing will help to highlight the central role of the food environment, i.e., the physical, economic, political and socio-cultural context in which each consumer engages with the food system, and its role in linking increased incomes with healthy and sustainable consumer food choices [50].

Gender is also a critical component of research and policy for FNS, given that women are considered to be nutrition ‘gatekeepers’, as income in the hands of women is more likely to be spent in ways that influence family nutrition and health outcomes, compared to income in the hands of men [51]. Women’s contributions to the fisheries/aquaculture sector, and the benefits derived from this contribution, is much broader than the specific focus taken in this review on the links between aquatic foods and FNS [52,53]. Recognition of the need for a gendered approach to fisheries and aquaculture has increased in research and development contexts in recent years [54,55]; however, regional and national fishery policies continue to contain broad and at times conflicting language around gender [56,57]. In terms of the links between gender, aquatic foods and FNS identified here, there was a focus on women as a vulnerable group, particularly within food, nutrition and health policies. An opportunity exists to extend recent policy approaches aimed at strengthening women’s leadership and economic empowerment in fisheries/aquaculture, to specifically improve the contribution of aquatic foods to FNS outcomes. Empowering women can improve nutrition outcomes [58] and will support their role as leaders for change toward healthy and sustainable food systems.

The least common context for linking FNS and aquatic foods was support for nutrition sensitive fisheries/aquaculture. This finding was not surprising given that current policies and public and private sector investments are most often framed around reducing poverty and food insecurity rarely through a nutrition-sensitive lens [5]. The term ‘nutrition-sensitive’ refers to policies and interventions that support improved nutrition outcomes [59] and is more established within agriculture. Support for a nutrition-sensitive approach to fisheries and aquaculture is warranted given the variation in nutrition profiles of aquatic foods and the implications for health outcomes [40,60,61]. The nutrition-sensitive approach to aquatic foods identified in the reviewed policies is promising, although further policy attention is required to determine how this approach can ensure aquatic foods help to address the crucial underlying determinants of nutrition [59]. Intersectoral collaboration will be critical to supporting this approach and to building coherence between sectoral policies. FNS is undermined by the lack of coordination and collaboration between the diverse sectors relevant to governance [62]. These linkages between governance arrangements are vital to meet national nutrition targets and maintain resilient food security systems.

While the sustainable management of natural resources, such as fisheries, is critical to their long-term contribution to food security, when it comes to governance arrangements for resilience, it is important to consider not only what is being made resilient to what, but also for whom [63]. Protecting habitats and species from human pressures, for example, is assumed to promote ecological communities that are more resilient [62]. However, whether this translates to improved food security more often remains inferred or theorised than empirically supported [61,64]. Conservation measures such as marine protected areas, in some cases, have been linked to the dispossession of land and marine access from coastal peoples [65] and the loss of property rights through ‘‘green/blue grabbing’’ [66], thereby reducing people’s access to marine resources, including food. More research is required to identify the opportunities to achieve multiple benefits, such as marine conservation, food security and climate action, at the national and global levels [11]. This research will also necessitate an understanding of the variation in both nutritional composition and environmental performance of diverse species caught or farmed using different methods [14].

In many of the policies reviewed, the risks of environmental degradation and climate change on fisheries/aquaculture was clearly identified, as well as measures to address the issues. Senegal has a large coastal population employed in primary production that is predicted to be negatively affected by climate change. As a result, there was a strong focus on the impacts of climate change on FNS and actions to be adopted to improve the resilience of the fisheries sector to avoid serious problems in the future. However, despite the potential impacts of climate change on aquatic foods and FNS, many instruments did not clearly articulate this link. National governments are beginning to include climate change explicitly in fisheries and aquaculture policies, e.g., [67]; however, further policy development is needed to ensure actions and indicators relating to climate change and stock management in particular, are more clearly aligned with FNS outcomes.

Education on the benefits of consuming aquatic foods, and how to prepare meals containing these foods, is an important aspect of increased consumption and improved nutrition [68]. A range of educational initiatives supporting the importance of aquatic food for human consumption were identified within the policies reviewed, with substantial differences between the policy approach of developed and developing countries. In the nutrition sector instruments of Japan and Norway, for example, the focus tended to be on the health benefits of nutritional compounds such as DHA in reducing depression, as well as on ensuring a balanced diet to prevent non-communicable diseases (NCDs) and obesity. In contrast, the policies of developing countries encourage the consumption of aquatic foods for protein, minerals and nutrients that support optimal growth and prevent malnutrition. The only country that simultaneously addressed the role of aquatic food in addressing diet-related NCDs and nutrition insecurity was Vanuatu, despite several countries reviewed facing the dual burden of over- and under-nutrition.

### 4.2. Current Best Practices

When identifying policy best practice for linking aquatic foods to food and nutrition security, a sole focus on the governance instruments of these specific sectors is too narrow. Fisheries, aquaculture and FNS all have inter-sectoral implications and were often dealt with in a comprehensive manner in multi-sectoral instruments outside their own sector, such as those relating to economic development, agriculture and climate change. For example, Bangladesh, Ghana and Tanzania had dedicated agricultural investment plans which made reference to the linkages between aquatic food and FNS to varying degrees. In the case of Bangladesh and Ghana, the plans covered a range of linkages, and in addition to specifying relevant objectives and actions they also assigned a budget to the actual objectives and/or actions. This additional step demonstrated commitment to taking action to address the matters identified and clarifying how targets will be met and actions implemented should be encouraged to strengthen the governance process.

While we can identify some foundational characteristics of best practice in terms of framing aquatic food and FNS policies, defining specific details of policy objectives requires further research. Best practices are not universally applicable because what works in one place will not necessarily be as effective in another [69], where national governments may face different nutritional, socio-economic, political, or environmental challenges. Differences in attention and approaches to these challenges were reflected in the instruments of some countries; for example, where a clear focus on a particular topic was evident across multiple documents. Taking a nutritional example, Chile has a relatively low per capita aquatic food consumption. Therefore, increasing consumption was the focus of the aquatic food and FNS linkages made in several of their documents. From a political perspective, in South Africa, which has historically lacked legal access rights for artisanal fishers, there was a strong focus on equitable access to fishery resources in several policy and planning documents. This approach to developing governance arrangements reflects the unique situation of the country and/or region, and ensures that these themes occur across a range of governance instruments. It is therefore important for coherence between sectors, including development policies, food security initiatives and the governance and development of the fisheries sector [70].

Potential examples of policy best practice from this review were The Second County Investment Plan 2016–2020 from Bangladesh, the Samoan Agriculture Sector Plan 2016–2020 (Volume 1 and 2) and the National Fisheries Sector Policy 2016–2031 for Vanuatu. Not only did they establish a connection between aquatic foods and FNS across a range of contexts (≥4), but they also showed a high level of political commitment through identified actions and targets that were clearly linked to the overarching aims and objectives of the policy. These instruments provide potential examples of food system policy framing that can be revised to suit different country contexts in future policy development and in reviews.

### 4.3. Reframing Policy for Food System Outcomes

While it is difficult to assess best practice from existing governance instruments, as this would require evidence of policy implementation and an evaluation of impact, which is beyond the scope of this study, it is possible, as this paper demonstrates, to identify policies that are well framed and which, therefore, offer useful guidance to the integration of FNS concerns with fisheries and aquaculture governance. We recommend some key attributes for future framing and development to shift policy towards supporting aquatic foods within the concept of a food system.

1. Broaden the contexts for the intersections between fisheries/aquaculture and FNS policies to look beyond developing fisheries/aquaculture to increase production, or looking only at increasing consumption, without consideration of supply and access. Considering the connection between fisheries/aquaculture and FNS through a range of different contexts within a policy, for example, increasing production and consumption and ensuring equitable and fair allocation of resources and distribution of benefits, will help to reframe the issue and is key to developing coherent food-systems based policies.
2. Support the link between fisheries/aquaculture and FNS across a range of both sectoral and multi-sectoral policies. This approach will help facilitate greater incorporation of fisheries and aquaculture into national food systems and food security dialogues and encourage cross-sectoral collaboration, which is necessary to manage the contribution of fisheries/aquaculture to a broad range of social, economic and environmental goals. Moving from a sectoral policy approach to fish and food, towards a multi-sectoral approach (e.g., across agriculture, fisheries, environment, public health nutrition, economic development) will further help establish a coherent food systems policy [71,72].
3. Include clear targets, actions and budget, as well as information on how the policy impact will be monitored and evaluated. In particular, it would be valuable to identify suitable metrics to assess FNS outcomes of fisheries/aquaculture-related activities as there was a clear lack of these in the documents reviewed. The assessment should consider which metrics provide the most meaningful insights into the actual impacts of changes to fisheries or aquaculture management, and related value chain activities, on FNS as well as the practicalities associated with collecting the necessary data. For example, new efforts such as the Food Insecurity Experience Scale (FAO, 2020d) is seeking to address the lack of consistency of tools to measure food security more generally and may be useful for future policy development in this field.
4. Strengthen support and awareness for nutrition-sensitive approaches to fisheries/aquaculture, which consider the underlying determinants of nutrition.

## 5. Conclusions

Aquatic foods play a vital role in providing healthy diets and livelihoods to millions of people around the world. Whilst numerous national governments articulate the connection between aquatic foods and FNS across a range of contexts in their governance approach, representing current ‘best practice’ in policy farming, further work is required to support the role of aquatic foods in FNS. Reframing polices to consider the connection between aquatic foods and healthy, fair and sustainable food outcomes, in combination with greater political support, will be a step toward developing more coherent food system-based policies.

## Figures and Tables

**Table 1:** List of countries selected for review based on the results of two-step prioritisation.

Country	Region	Prioritisation Step 1	Prioritisation Step 2	
			Aquatic Food Policy Link with FNS (4 = High, 1 = Low) [17]	FNS Policy Link with Aquatic Food (4 = High, 1 = Low) [17]
Bangladesh	South Asia	Large aquatic food producer	4	4
Chile	South America	Large aquatic food producer	1	3
Ghana	West Africa	Reliance on aquatic foods	1	3
India	South Asia	Large aquatic food producer	3	0
Indonesia	South East Asia	Large aquatic food producer	NA	2
Japan	East Asia	Large aquatic food producer	3	1
Mauritania	West Africa	Large aquatic food producer	4	3
Norway	Europe	Large aquatic food producer	0	3
Peru	South America	Large aquatic food producer	4	3
Philippines	Southeast Asia	Large aquatic food producer	3	0
Samoa	Polynesia	Reliance on aquatic foods	3	0
Senegal	West Africa	Reliance on aquatic foods	2	2
South Africa	Africa	Large aquatic food producer	3.5	1
Tanzania	Africa	Reliance on aquatic foods	3	1
Vanuatu	South Pacific	Reliance on aquatic foods	4	3

NA, not available.

**Table 2:** Context of linkage between FNS and fisheries/aquaculture.

**A**	Develop fisheries/aquaculture sector to increase aquatic food availability and/or access
**B**	Develop fisheries/aquaculture sector as a livelihoods approach to improve food security
**C**	Support a nutrition sensitive approach to fisheries/aquaculture
**D**	Improve sustainability/resilience of fisheries/aquaculture systems to protect long term food security and/or livelihoods
**E**	Ensure equitable and fair allocation of resources and distribution of benefits to improve food security and/or improve livelihoods
**F**	Increase aquatic food consumption
**G**	Support contribution of aquatic food to diets and/or livelihoods of vulnerable groups (children, women, rural, poor)
**H**	Education on the nutritional benefits of aquatic food and/or guidance on utilisation
**I**	Encourage cross-departmental collaboration

**Table 3:** Classification of the level of political commitment.

Level of Commitment	Description
None	No mention of linkage
Very low	Search terms appear in general discussions
Low	Search terms stated in general aims of the instrument but not linked to clear objectives
Moderate	Search terms linked to objectives, but no details provided on implementation or measurement
High	Search terms are linked to objectives with clear details on how the objective will be implemented OR search terms are linked to objectives with targets set to measure performance
Very high	Search terms are linked to objectives with clear details on how the objective will be implemented AND targets set to measure performance

**Table 4:** Summary of sectors for documents reviewed.

Sector	Count	% Total
Agriculture	11	10%
Aquaculture	11	10%
Climate change	5	5%
Economic Development	8	7%
Financial investment	4	4%
Fisheries	32	29%
Fisheries and Aquaculture	6	5%
Food security	5	5%
Food security and nutrition	8	7%
Health	1	1%
Natural resource management	1	1%
Nutrition	11	10%
Social development	3	3%
Sustainable Development	4	4%

**Table 5:** Summary of linkages made between aquatic food and FNS.

Context of Linkage	Count	% Total	Example
Develop fisheries/aquaculture sector to increase aquatic food availability and/or access	56	51%	Ghana’s National Nutrition Policy 2013–2017 objectivises 3 aims to promote practices that will ensure availability, access, diversity, proper storage and utilisation of a variety of foods, including practices that encourage a increased and diversified food crop, livestock and fisheries production
Develop fisheries/aquaculture sector as a livelihoods approach to improve food security	36	33%	The Indian National Policy for Farmers 2007 describes reforms to ensure access to productive assets (e.g., fishpond) in order to increase household income, to ensure nutrition and livelihood security
Improve sustainability/resilience of the system to protect long term food security and/or livelihoods	29	26%	Vanuatu’s National Plan of Action on Food and Nutrition Security 2013–2015 lists improved resilience of agriculture, livestock and fisheries production systems as an outcome of an objective to enhance the sustainable production, processing, trading, marketing and use of safe and nutritious foods
Education on the nutritional benefits of aquatic food and/or guidance on utilisation	16	15%	Japan’s Basic Act on Dietary Education (Shokuiku) 2005 encourages collaboration between educators and farmers/fishers to provide educational opportunities for the general population to better understand the importance of human activities in food production and distribution
Increase aquatic food consumption	15	14%	Indonesia’s National Mid-term Development Plan 2020–2024 (Rencana Pembangunan Jangka Menengah Nasional 2020–2024) includes a development achievement of increasing fish consumption to 47.3 kg/capita/year
Ensure equitable and fair allocation of resources and distribution of benefits to improve food security and/or improve livelihoods	12	11%	Policy for the Small-Scale Fisheries Sector in South Africa 2012 outlines that the state must promote equitable access to and involvement in all aspects of the fishery, in particular noting past prejudice against women and other marginalised groups
Support contribution of aquatic food to diets and/or livelihoods of vulnerable groups (children, women, rural, poor)	11	10%	National Agricultural Investment Program for Food Security and Nutrition in Senegal 2018–2022) specifically address women and youth employed in fisheries and aquaculture
Encourage cross-departmental collaboration	11	10%	Bangladesh’s National Fisheries Strategy 2006 states the Department of Fisheries should collaborate with other Departments, government agencies, non-governmental and commercial organisations to establish networks and forums for interchange of M&E data in the areas of nutrition (supply of animal protein) and public health.
Support a nutrition sensitive approach to fisheries/aquaculture	6	5%	Bangladesh’s Second Country Investment Plan: nutrition-sensitive food systems (2016–2020) includes a priority intervention to improve management of fisheries, livestock and poultry to increase production and productivity and nutritional value while ensuring sustainability
No mention of the linkage	38	35%	NA

**Table 6:** Summary of the level of commitment to linking aquatic food and FNS.

Level of Commitment	Description	Count	% Total
None	No mention of linkage	38	35%
Very low	Search terms appear in general discussions	7	6%
Low	Search terms stated in general aims of the instrument but not linked to clear objectives	21	19%
Moderate	Search terms linked to objectives, but no details provided on implementation or measurement	15	14%
High	Search terms are linked to objectives with clear details on how the objective will be implemented OR Search terms are linked to objectives with targets set to measure performance OR budget allocated	16	15%
Very high	Search terms are linked to objectives with clear details on how the objective will be implemented AND targets set to measure performance OR budget allocated	13	12%

**Table 7:** Linkages and high level of commitment by country.

Country	Number of Documents Examined	Instruments with Linkage	Instruments with 3 or More Policy Frames	Instruments with High/Very High Commitment
Indonesia	10	90% (N = 9)	30% (N = 3)	40% (N = 4)
Tanzania	9	89% (N = 8)	33% (N = 3)	11% (N = 1)
Vanuatu	7	86% (N = 6)	43% (N = 3)	57% (N = 4)
Senegal	6	83% (N = 5)	50% (N = 3)	17% (N = 1)
Bangladesh	14	79% (N = 11)	43% (N = 6)	36% (N = 5)
Samoa	8	75% (N = 6)	63% (N = 5)	38% (N = 3)
Ghana	10	70% (N = 7)	20% (N = 2)	30% (N = 3)
Mauritania	6	67% (N = 4)	0% (N = 0)	17% (N = 1)
South Africa	9	56% (N = 5)	22% (N = 2)	22% (N = 2)
Peru	6	50% (N = 3)	33% (N = 2)	17% (N = 1)
Philippines	4	50% (N = 2)	25% (N = 1)	0% (N = 0)
India	5	40% (N = 2)	40% (N = 2)	20% (N = 1)
Norway	3	33% (N = 1)	0% (N = 0)	33% (N = 1)
Chile	7	29% (N = 2)	0% (N = 0)	29% (N = 2)
Japan	5	20% (N = 1)	0% (N = 0)	0% (N = 0)

## Supplementary Materials

The following are available online at https://www.mdpi.com/article/10.3390/foods10071589/s1, Table S1: FNS terms used to search fisheries/aquaculture governance instruments, Table S2: Fisheries/aquaculture terms used to search food security/food security documents, Table S3 Summary of governance instruments and the linkages between fisheries/aquaculture and FNS.
