# Supplementary material for: Identifying Policy Best-Practices to Support the Contribution of Aquatic Foods to Food and Nutrition Security
Source: Foods. 2021 Jul 8;10(7):1589. doi: 10.3390/foods10071589 (PMC8303926; doi:10.3390/foods10071589)
Supplement: Supplementary file 1 [file foods-10-01589-s001.zip › foods-1223804-supplementary.pdf]

Identifying policy best-practices to support the contribution of aquatic foods to food security and nutrition

AK Farmery, A White, EH Allison

Table S1: FNS terms used to search fisheries/aquaculture governance instruments

English	Spanish	French	Bahasa Indonesian
Food security	seguridad alimentaria	La sécurité alimentaire	Ketahanan Pangan
Nutrition	nutrición; alimentación	La nutrition	Nutrisi
Malnutrition	malnutrición; desnutrición	La malnutrition	Malnutrisi
(Food) access	acceso (a alimento, a alimentación, alimentaria)	L'accès/ l'accessibilité (aux aliments)	Akses ke makanan
(Food) availability	disponibilidad	La disponibilité (des aliments)	Ketersediaan makanan
(Food) utilization	utilización biológica	L'utilisation (des aliments)	Penggunaan makanan
Affordable (food)	asequible	Le prix abordable (des aliments)	terjangkau
Hunger	hambre	La faim	Kelaparan
Livelihood	medios de vida; sustento	Les moyens de subsistance	Kebutuhan hidup
Poverty	pobreza	La pauvreté	Kemiskinan

Table S2: Fisheries/aquaculture terms used to search food security/food security documents

English	Spanish	French	Bahasa Indonesian
Fish	pez; peces; pescado	Le poisson	Ikan
Fisheries	pesquería	La pêche	Perikanan
Aquaculture	acuicultura	L'aquaculture	Budidaya Laut
Seafood	mariscos	Les fruits de mer	Makanan Laut
Mariculture	maricultura	La mariculture	Kultur Maritim

Table S3 Summary of governance instruments and the linkages between fisheries/aquaculture and FNS

Country	Source document	Sector	Linkage between fish and FSN	Context of Linkage (see Table S4)	Level of Commitment
Bangladesh	National Fisheries Strategy 2006	Fisheries	Yes	B,I	Very low
Bangladesh	National Fisheries Policy 1998	Fisheries	Yes	A	Very low
Bangladesh	Inland Capture Fisheries Sub-Strategy 2006	Fisheries	No	NA	None
Bangladesh	Marine Fisheries Sector Sub-Strategy 2006	Fisheries	No	NA	None
Bangladesh	Shrimp Sub-Strategy 2006	Fisheries and Aquaculture	Yes	B	Very low
Bangladesh	Coastal Development Strategy 2006	Natural Resource Management	Yes	A,B,D,E	Moderate
Bangladesh	National Aquaculture Development Strategy and Action Plan 2013–2020	Aquaculture	Yes	A,B,D,E,G	Very high
Bangladesh	National Nutrition Policy 2015	Nutrition	Yes	A,C,H,I	Moderate
Bangladesh	National Food Policy 2006	Food security	Yes	A,B	High
Bangladesh	National Food Policy Plan of Action 2008-2015	Food security	Yes	A,B,F,H,I	Very high
Bangladesh	Protection and Conservation of Fish Act 1950	Fisheries	No	NA	None
Bangladesh	Perspective Plan of Bangladesh 2010-2021	Agriculture	Yes	A,C,H	Moderate
Bangladesh	Second Country Investment Plan (2016-2020)	Financial investment	Yes	A,B,C,D,E,F,J,H,I	Very high
Bangladesh	Seventh Five Year Plan (2016-2020)	Food security and nutrition	Yes	A,B	High
Chile	National Fisheries Policy 2007	Fisheries	No	NA	None
Chile	Chilean Action Plan for Sustainable Consumption and Production 2017-2022	Sustainable Development	Yes	F	Very high
Chile	National Health Strategy 2011-2020	Health	No	NA	None
Chile	NA	Aquaculture	No	NA	None
Chile	Climate Change Adaptation Plan for the Health Sector 2016	Climate change	No	NA	None
Chile	Climate Change Adaptation Plan for Fisheries and Aquaculture 2015	Climate change	Yes	F	High

Chile	General Law of Fishing and Aquaculture 1998	Fisheries and aquaculture	No	NA	None
Ghana	Fishery Management Plan of Ghana 2015-2019	Fisheries	Yes	A,D	Low
Ghana	National Nutrition Policy 2013-2017	Nutrition	Yes	A,C,I	Moderate
Ghana	Ghana National Aquaculture Development Plan 2012	Aquaculture	Yes	A	Low
Ghana	National Climate-Smart Agriculture and Food Security Action Plan 2016-2020	Climate change	Yes	B,D	High
Ghana	The Coordinated Programme of Economic and Social Development Policies 2017-2024	Social development	No	NA	None
Ghana	Medium-term National Development Policy Framework 2018-2021	Economic Development	No	NA	None
Ghana	Medium-term Agricultural Sector Investment Plan II, 2014-2017	Financial investment	Yes	C,H,G,	High
Ghana	National Medium-Term Development Plan of Ministry of Food and Agriculture 2014-2017	Economic Development	Yes	A	Very high
Ghana	Ghana Shared Growth and Development Agenda 2014-2017	Financial investment	Yes	A,B	Moderate
Ghana	Fisheries Act 2002	Fisheries	No	NA	None
India	National Policy on Marine Fisheries 2017	Fisheries	Yes	A,B,G	Low
India	National Policy for Farmers 2007	Agriculture	Yes	A,B,E	High
India	National Nutrition Strategy 2017	Nutrition	No	NA	None
India	The National Food Security Law 2013	Food security	No	NA	None
India	The Indian Fisheries Act 1897	Fisheries	No	NA	None
Indonesia	Bill of the Republic of Indonesia Number 18 Year 2012 Concerning Food by the Mercy of God Almighty	Food security and nutrition	Yes	D,F	Low
Indonesia	National Plan of Action for Food and Nutrition 2011-2015	Nutrition	Yes	A,F	Very high
Indonesia	Strategic Planning Document of the Maritime and Fisheries Department of Central Java Province 2018-2023	Fisheries	Yes	A,H	Very high
Indonesia	Strategic Planning Document of the Maritime and Fisheries Department of Lampung Province 2019-2024	Fisheries	Yes	A,H	Very high
Indonesia	Strategic Planning Document of the Maritime and Fisheries Department of South Kalimantan Province 2018-2023	Sustainable Development	Yes	B,E	Moderate

Indonesia	Strategic Planning Document of the Maritime and Fisheries Department of West Java Province 2018-2023	Fisheries	Yes	D,F,H	Very high
Indonesia	Strategic Planning Document of the Maritime and Fisheries Department of Riau Province 2018-2023	Fisheries	Yes	A,D,F	Moderate
Indonesia	Strategic Planning Document of the Maritime and Fisheries Department of Nusa Tenggara Barat Province 2018-2023	Fisheries	Yes	D,F	Low
Indonesia	National Mid-term Development Planning 2020-2024	Social development	Yes	A,B,D	Moderate
Indonesia	Fisheries Law No.31/2004	Fisheries	No	NA	None
Japan	Basic Act on Dietary Education (Shokuiku) 2005	Nutrition	Yes	H	Low
Japan	Climate Change Adaptation Plan of Ministry of Agriculture, Forestry and Fisheries 2015	Climate change	No	NA	None
Japan	Basic Policy and Action Plan for the Revitalization of Japan's Food Agriculture, Forestry and Fisheries 2011	Agriculture	No	NA	None
Japan	Sustainable Aquaculture Production Assurance Act 1999	Aquaculture	No	NA	None
Japan	Fisheries Basic Act 2001	Fisheries	No	NA	None
Mauritania	National Responsible Management Strategy for Sustainable Development of Fisheries and Maritime Economy 2015-2019	Fisheries and Aquaculture	Yes	A,H	High
Mauritania	Multisectoral Strategic Nutrition Plan 2016-2025	Nutrition	Yes	A	Moderate
Mauritania	National Strategy for Accelerated Growth and Shared Prosperity (SCAPP 2016-2030), Volume II	Economic Development	Yes	H	Low
Mauritania	National Food Security Strategy for Mauritania for 2015 and Vision 2030	Food security	No	NA	None
Mauritania	National Social Protection Strategy in Mauritania 2012	Social development	Yes	A	Very low
Mauritania	Law N ° 2000-025 / on the Fisheries Code	Fisheries	No	NA	None
Norway	Marine Resources Act 2008	Fisheries	No	NA	None
Norway	National Action Plan for a Healthier Diet 2017	Nutrition	Yes	D,F	Very high
Norway	Strategy for an Environmentally Sustainable Norwegian Aquaculture Industry 2009	Aquaculture	No	NA	None
Norway	Aquaculture Act 2005	Fisheries	No	NA	None
Peru	National Aquatic Development Plan 2010 - 2021	Aquaculture	Yes	A,B,E	Very low
Peru	National Plan for the Development of Artisanal Fishing 2004	Fisheries	Yes	F,H,I	High

Peru	The Multiannual Sector Strategic Plan 2015-2021	Agriculture	No	NA	None
Peru	The National Plan for Food and Nutrition Security 2015-2021	Food security and nutrition	No	NA	None
Peru	Law No. 27460 - Law for the Promotion and Development of Aquaculture 2001	Aquaculture	No	NA	None
Peru	Decree Law No. 25977 - General Fishing Law 1992	Fisheries	Yes	A,B	Low
Philippines	Comprehensive National Fisheries Industry Development Plan 2006-2025	Fisheries and Aquaculture	Yes	A,B	Low
Philippines	Fisheries Code 1998	Fisheries and Aquaculture	Yes	A,B,D,E	Low
Philippines	Philippine Plan of Action for Nutrition 2017-2022	Nutrition	No	NA	None
Philippines	Philippine Development Plan 2017-2022	Economic Development	No	NA	None
Samoa	Samoa Tuna Management and Development Plan 2011-2015	Fisheries	Yes	E	Low
Samoa	National Food and Nutrition Policy 2013	Food security and nutrition	No	NA	None
Samoa	Samoa Coastal Fisheries Management Plan 2013-2016	Fisheries	Yes	A,B,D	High
Samoa	Aquaculture Management and Development Plan 2013-2016	Aquaculture	Yes	A,B,D	Low
Samoa	Agriculture Sector Plan 2016-2020 - Volume 1	Agriculture	Yes	A,B,D,F,G	Very high
Samoa	Agriculture Sector Plan 2016-2020 - Volume 2	Agriculture	Yes	A,B,D,F,G	Very high
Samoa	Strategy for the Development of Samoa 2016-2020	Economic Development	Yes	A,B,D	Moderate
Samoa	Fisheries Act 1988	Fisheries	No	NA	None
Senegal	Maritime Fisheries Code 1998	Fisheries	No	NA	None
Senegal	National Agricultural Investment Program for Food Security and Nutrition in Senegal 2018-2022	Food security and nutrition	Yes	A,D,G,I	High
Senegal	National Nutrition Development Policy, 2015-2025	Nutrition	Yes	A,H	Low
Senegal	National Strategy for Marine Protected Areas (MPAs) of Senegal 2013	Fisheries	Yes	A	Low

Senegal	National Strategy for Food Security and Resilience (SNSAR, 2015-2035)	Food security	Yes	A,B,D,G,I	Moderate
Senegal	National adaptation plan for the fisheries and aquaculture sector in the face of climate change by 2035	Climate change	Yes	A,B,D	Low
South Africa	Policy for the small-scale fisheries sector in South Africa 2012	Fisheries	Yes	A,B,D,E,I	Moderate
South Africa	Aquaculture and Economic Development Awareness Strategy for South Africa 2012-2016	Aquaculture	No	NA	None
South Africa	National Aquaculture Policy Framework for South Africa 2013	Aquaculture	Yes	A,B,E	High
South Africa	National Policy on Food and Nutrition Security 2013	Food security and nutrition	No	NA	None
South Africa	Agricultural Policy Action Plan 2015-2019	Agriculture	Yes	A,B	Low
South Africa	Strategic Plan for the Department of Agriculture, Forestry and Fisheries 2013/14-2017/18	Agriculture	Yes	A,B	High
South Africa	National Development Plan 2030	Economic Development	Yes	A	Very low
South Africa	Roadmap for Nutrition in South Africa 2013-2017	Food security and nutrition	No	NA	None
South Africa	Marine Living Resources Act No 18 of 1998	Fisheries	No	NA	None
Tanzania	National Fisheries Policy of 2015	Fisheries	Yes	A,B	Low
Tanzania	National Nutrition Strategy 2011/12-2015/16	Nutrition	Yes	A	Very low
Tanzania	Agricultural Sector Development Programme Phase Two 2016	Agriculture	Yes	A,B,D,G,H	Low
Tanzania	National Multisectoral Nutrition Action Plan 2016/17-2020/21	Nutrition	Yes	A,C,D,F,G,H,I	Low
Tanzania	National Five-Year Development Plan 2016/17-2020/21	Economic Development	Yes	A	High
Tanzania	Agricultural Sector Development Strategy II 2015/16 - 2024/25	Agriculture	Yes	A	Moderate
Tanzania	National Strategy for Growth and Reduction of Poverty 2010	Economic Development	Yes	A,B	Moderate
Tanzania	Tanzania Agriculture and Food Security Investment Plan 2011/12 - 2020/21	Financial investment	Yes	A,B,D,G	Low

Tanzania	Fisheries Act 2010	Fisheries	No	NA	None
Vanuatu	Vanuatu Aquaculture Development Plan 2008-2013	Aquaculture	Yes	A,B	Low
Vanuatu	Vanuatu National Fisheries Sector Policy 2016-2031	Fisheries and Aquaculture	Yes	A,B,D,F	Very high
Vanuatu	National Plan of Action on Food and Nutrition Security 2013-2015	Food security and nutrition	Yes	A,B,D,E,G,I	High
Vanuatu	Overarching Productive Sector Policy 2012-2017	Agriculture	Yes	A,B,D,E	Moderate
Vanuatu	National Sustainable Development Plan NSDP 2016 to 2030	Sustainable Development	Yes	A,D	High
Vanuatu	National Sustainable Development Plan 2016 to 2030 - Monitoring and Evaluation Framework	Sustainable Development	Yes	A,D	High
Vanuatu	Fisheries Act No 10	Fisheries	No	NA	None
